# Understanding the Association of Self-Efficacy, Mood, and Demographics with Physical Activity in Syrian and Iraqi Refugees: A Cross-Sectional Study in Jordan

**DOI:** 10.1155/2023/8876254

**Published:** 2023-09-19

**Authors:** Rula A. Amr, Ahmed M. Al-Smadi, Rula A. Deiranieh, Romel A. Amr, Amal H. Mayyas, Rand T. Akasheh

**Affiliations:** ^1^Department of Nutrition and Dietetics, American University of Madaba, Madaba, Jordan; ^2^Department of Adult Health Nursing, Al Al-Bayt University, Mafraq, Jordan; ^3^Department of Nutrition and Food Processing, Balqa Applied University, Salt, Jordan; ^4^Department of Trauma and Orthopaedic, St George's University Hospital, London, UK; ^5^Department of Pharmacy, American University of Madaba, Madaba, Jordan; ^6^Department of Internal Medicine, The Ohio State University, Columbus, Ohio, USA

## Abstract

**Objective:**

This cross-sectional study aimed at investigating the influence of sociodemographic factors on physical activity among Syrian and Iraqi refugees in Jordan. In addition, it sought to determine the predictive ability of self-efficacy and mood in relation to the level of physical activity in this population.

**Methods:**

A convenient sample of refugees residing in Jordanian cities was collected. Participants completed a self-administered questionnaire pack consisting of a demographic data sheet, a physical activity level questionnaire, the Brunel Mood Scale, and the General Self-Efficacy Scale. Descriptive analysis was used to analyze demographic details, while the chi-square test examined the association between physical activity and demographic factors. The independent *t*-test assessed differences in self-efficacy and mood subscales in relation to physical activity. Logistic regression analysis was employed to identify potential predictors of the two categories of physical activity.

**Results:**

Most participants reported low levels of physical activity. The frequency of moderate-to-high physical activity was higher in male participants, those with higher education, better health, and higher income. Compared to participants of low physical activity, those in the moderate-to-high physical activity category expressed significantly higher mean score of self-efficacy but lower mean scores of tension, depression, anger, vigor, fatigue, and confusion, indicating better mood. The logistic regression analysis for physical activity indicated that the model was significant for education, income, good health perception, self-efficacy, and one mood subscale (vigor), with these variables collectively accounting for 11–18% of the variance (*P* value <0.001).

**Conclusion:**

The higher physical activity level is significantly associated with being male, higher education, higher income, better health, higher self-efficacy, and increased vigor. These findings highlight the importance of considering sociodemographic factors and psychological aspects, such as self-efficacy and mood, when addressing physical activity among refugees in Jordan.

## 1. Introduction

The influx of Syrian and Iraqi nationals seeking refuge in neighboring countries has been steadily increasing due to political and ethnic conflicts [1]. In Jordan, a significant number of Syrian and Iraqi refugees have sought asylum, with approximately 1.4 million Syrians and 58,050 Iraqis registered with the United Nations High Commission for Refugees in 2015 [2]. These refugees face numerous challenges, including high unemployment rates, economic insecurity, and inadequate living conditions in housing facilities provided by nongovernmental organizations [3].

The economic and social changes experienced by Syrian and Iraqi refugees have had a profound impact on their way of life [4, 5]. The adoption of a sedentary lifestyle has become prevalent among the refugee population, increasing their susceptibility to obesity, type II diabetes, and cardiovascular disorders. Promoting a healthier and active lifestyle is crucial for the well-being of both refugees and their host nations [6, 7]. Global research indicates a significant increase in the prevalence of overweight and obesity, particularly among children, who are at risk of carrying excess body fat into adulthood [8].

In Jordan, a host country with limited resources, priority is given to the treatment and care of chronic conditions, communicable diseases, and mental health disorders [9]. Consequently, resources dedicated to improving physical activity and sports participation among refugees are often lacking. Refugees experience high levels of stress, which is a known risk factor for various mental disorders [10] and strongly correlates with poor mental and physical health [11], thereby impacting their quality of life [2].

Although physical activity guidelines have been established to promote better health and quality of life, many adults struggle to maintain high levels of physical activity, which becomes increasingly challenging with age [12]. Individuals with chronic diseases often find it overwhelming to make changes to their activity levels [12].

Self-efficacy, a crucial psychosocial factor, plays a significant role in determining physical activity behavior. Rooted in the cognitive theory, self-efficacy reflects an individual's belief in their ability to accomplish specific goals and missions [13]. It empowers individuals to overcome barriers, invest effort and time, and attain their desired objectives. Self-efficacy has a substantial impact on the adoption of physical activity [14].

To the best of our knowledge, no previous study has examined the association between sociodemographic variables, physical activity, and self-efficacy specifically in Syrian and Iraqi refugees in Jordan. Therefore, this study aims to address this research gap and investigate the predictive abilities of mood and self-efficacy on physical activity levels within this population.

## 2. Methods

### 2.1. Design

This study employed a secondary analysis of a previously published cross-sectional study by Amr et al. [1] to investigate the association between physical activity and demographic variables, self-efficacy, and mood among Iraqi and Syrian refugees in Jordan. In addition, logistic regression analysis was conducted to identify potential predictors of the two categories of physical activity in this population.

### 2.2. Sample Size

Power analysis was conducted on the basis of an estimated population size of one million, a confidence level of 95%, and a confidence interval of 3, and it indicated that a minimum sample size of 1030 participants is required for this study.

### 2.3. Participants

Convenience sampling was used to recruit Iraqi and Syrian refugees living in Jordan. The study enrolled a total of 1038 participants. Adults aged 18 and up who had been living in Jordan as refugees for at least two months met the inclusion criteria. There were no withdrawals from the study.

### 2.4. Setting

Participants were recruited from four Catholic churches situated in the Jordanian cities of Amman, Madaba, and Zarqa. These locations served as the primary settings for data collection. Unfortunately, we did not have access to refugee camps or enough resources to include refugees from these sites in the study.

### 2.5. Ethical Consideration

The current study was conducted in accordance with the Helsinki Declaration guidelines. The Ethical Committee of the American University of Madaba (AUM) approved all procedures related to this study. All participants provided written informed consent before data collection.

### 2.6. Data Collection

Participants were recruited from designated church facilities providing accommodation for refugees. The data collection period occurred over a span of three months starting from September 2017 and was conducted by trained graduate students from the Department of Nutritional Sciences at AUM. Prior to participation, each participant received a comprehensive explanation of the study's objectives and procedures. In addition, informed consent was obtained from all participants through the signing of a consent form. Participants who encountered challenges related to reading comprehension were offered assistance to ensure accurate completion of the questionnaire. On average, participants required approximately 30–40 minutes to complete the questionnaire.

### 2.7. Instruments

A self-administered questionnaire pack written in Arabic was utilized to collect data in this study. The questionnaire pack encompassed several instruments, including a demographic data sheet, a physical activity-level questionnaire, the Arabic version of the Brunel Mood Scale (BRUMS), and the General Self-Efficacy Scale (GSE). The demographic and clinical information obtained from participants encompassed variables such as age, gender, educational level, marital status, employment status, number of family members, medication availability, and presence of chronic illness.

To assess participants' mood, the Arabic version of the BRUMS was employed. The BRUMS scale consists of 24 items that measure six distinct mood states (tension, depression, anger, vigor, fatigue, and confusion) [15], whereby each mood state is represented by a subscale consisting of 4 items. Respondents rate a list of adjectives based on their emotional states during the previous week or at the time of evaluation, on a 5-point Likert scale ranging from 0 to 4 (0 = not at all; 1 = a bit; 2 = moderate; 3 = enough; and 4 = extremely). The scores for each subscale range from 0 to 16, and individual scales are assessed separately, although they are interrelated [15].

The General Self-Efficacy Scale (GSE), a self-report measure of self-efficacy, was employed in this study [16]. The GSE consists of 10 items and is associated with emotions, optimism, and work satisfaction. Negative coefficients have been found for depression, stress, health complaints, burnout, and anxiety. The scale assesses an individual's general sense of perceived self-efficacy and aims to predict coping with daily challenges and adaptation after experiencing stressful events. The total score is calculated by summing all the item responses. For the GSE, the total score ranges from 10 to 40, with higher scores indicating greater self-efficacy [16].

The questionnaire also included inquiries about participants' weekly physical activity levels. Physical activity was classified according to the guidelines of the 2005 International Physical Activity Questionnaire [17]. High-level physical activity was defined as engaging in vigorous physical activity for more than three days a week at an exercise level of 1,500 metabolic equivalent (METs) or performing more than 3,000 METs of exercise level, including walking and moderate or vigorous-intensity exercise, for more than seven days a week. Moderate-level physical activity was defined as engaging in intense activity for more than 20 minutes on more than three days a week, or performing moderate-intensity exercise, or walking for more than 30 minutes each time and more than five days a week, or achieving 600 METs of exercise level with walking, or activity of moderate or vigorous intensity. Low-level physical activity was defined as not falling into either of the two aforementioned groups [18].

### 2.8. Data Analysis

The Statistical Package for the Social Sciences (SPSS) software version 21 was used for data analysis. The demographic variables were analyzed using descriptive analysis. The chi-square test was used to examine the association between physical activity and demographic variables. In addition, the independent samples *t*-test was used to examine the differences in self-efficacy and mood across physical activity categories. This was followed by logistic regression to examine the possible predictors of the two categories of physical activity.

## 3. Results

### 3.1. Demographic Characteristics

Table 1 presents the demographic characteristics of the participants. The majority of participants fell within the age range of 18–30 years (*n* = 477, 46%), were married (*n* = 809, 77.9%), identified as female (*n* = 737, 61.4%), had primary school education (*n* = 306, 29.5%), were unemployed (*n* = 904, 78.1%), reported having chronic diseases (*n* = 609, 58.7%), were of Syrian nationality (*n* = 826, 79.6%), rated their health as good (*n* = 625, 60.2%), were nonsmokers (*n* = 789, 76%), and perceived their income as poor (*n* = 937, 90.3%).

### 3.2. Physical Activity, Self-Efficacy, and Mood

Regarding physical activity, the majority of participants exhibited low physical activity levels (*n* = 841, 81%), while 156 participants reported moderate physical activity (15%) and 41 participants engaged in high physical activity (3.9%). However, for the purpose of logistic regression analysis, physical activity was dichotomized into low vs. moderate-to-high categories. The descriptive analysis of the self-efficacy scale indicated a mean score of 25.94 (SD = 6.20) ranging from 18 to 89. The descriptive analysis for each mood subscale revealed the following mean scores: anger 7.37 (SD = 4.23), confusion 7.28 (SD = 4.65), depression 6.57 (SD = 4.32), fatigue 7.05 (SD = 4.17), tension 7.72 (SD = 4.06), and vigor 8.53 (SD = 4.57), with scores ranging from 0 to 16 for each subscale.

### 3.3. Differences in Self-Efficacy and Mood Subscales across Physical Activity Levels

The independent *t*-test revealed significant differences in self-efficacy and each mood subscale among participants with different physical activity levels, as shown in Table 2. The mean self-efficacy score was significantly higher in participants who were moderately to highly active compared with low active participants. Conversely, mean scores for anger, confusion, depression, fatigue, tension, and vigor were all significantly lower in participants in the moderate-to-high physical activity category compared with those in the low physical activity category.

### 3.4. Differences in Physical Activity Based on Demographic Variables

The associations between demographic variables and physical activity were assessed using the chi-square test. Table 1 presents the results, indicating that a higher percentage of male participants had moderate-to-high physical activity levels compared with females (*P* = 0.004). The proportion of participants with moderate-to-high physical activity increased with higher education levels compared with lower levels of education (*P* = 0.001). A higher percentage of participants reporting very good health were in the moderate-to-high physical activity category compared to those perceiving their health as good or bad (*P* = 0.005). Furthermore, a higher percentage of participants who perceived they had enough were moderately to highly active compared to those reporting not enough income (*P* = 0.001).

### 3.5. Predictors of Physical Activity

Logistic regression analysis was performed to identify potential predictors of the two categories of physical activity. Only factors that showed significant differences or associations with physical activity were included as predictors: gender, education, sufficient income, health status, self-efficacy, and the mood subscales.  Table 3 presents the results of the logistic regression analysis, indicating that the model was significant (*P* = 0.001) and accounted for 11–18% of the variance. The significant predictors of higher physical activity levels among participants were male gender, higher education, sufficient income, good health status, higher self-efficacy, and higher vigor. These findings suggest that being educated, having enough income, good health, higher self-efficacy, and higher vigor are all predictors of higher physical activity levels.

## 4. Discussion

The study population consisted primarily of Syrians, comprising approximately 80% of the participants, who reported low levels of physical activity. It is well established that refugees, including the present study's population, face challenges in maintaining an active lifestyle comparable to their home countries. The findings of this study revealed a significant disparity in activity levels between genders, with approximately 23% of males exhibiting moderate-to-high activity levels compared with only 16% of females. This gender difference aligns with the results reported by Teresa M Bianchini de Quadros et al. in their 2009 survey, which investigated sociodemographic factors influencing the physical activity and found higher levels of inactivity among females [19]. Several factors may contribute to this disparity, including the social environment in which women live, which may be less conducive to physical activity compared with men. In addition, Muslim women refugees encounter various obstacles and difficulties in adopting active lifestyles due to traditional cultural norms. Female refugees often bear the responsibility of caring for family members, including children and other women, further complicating their ability to engage in regular physical activity. Consequently, maintaining adequate levels of physical activity poses significant challenges for females in this vulnerable population, potentially accounting for the observed gender differences. Notably, it has been observed that men tend to overestimate their level of physical activity participation compared with women [20].

Intriguingly, participants with higher education levels (secondary school and university) demonstrated greater levels of physical activity compared with those with lower educational attainment (participants below secondary levels). These findings are consistent with previous research. For instance, Kaplan et al. investigated a cohort of 12,611 Canadian individuals aged 65 years and above, recruited from the Canadian National Population Health Survey, and identified several factors associated with habitual physical activity, including gender (male), younger age, higher education levels, absence of chronic conditions, and lower body mass index. Interestingly, they also reported a positive correlation between lower psychological distress, as measured by the Generalized Distress Scale, and habitual physical activity [21]. Similarly, Kaplan et al. [21] found that a greater number of participants engaged in moderate-to-high activity levels among those reporting sufficient income. Our study's findings align with previous research conducted in Korea, where individuals residing in low-income households faced greater challenges in engaging in physical activity compared with those in higher-income households. This disparity can be partly attributed to environmental and social barriers, such as limited access to sports facilities, transportation services, and free time constraints [22]. In Jordan, Syrian and Iraqi refugees residing in crowded housing camps with limited access to sports and recreational facilities further exemplify the impact of socioeconomic status on physical activity levels. Importantly, many studies have reported that socioeconomic status (SES) correlates with physical activity [23, 24]. Factors such as engagement in manual and industrial labor, lower income, lower literacy, and educational levels have consistently been associated with lower levels of physical activity [24]. Various explanations have been proposed for the lower physical activity levels observed among individuals with low socioeconomic status. These include limited availability of parks and recreational facilities in their communities, financial constraints hindering the purchase of home exercise equipment, lack of social encouragement and support for leading physically active lifestyles, and limited knowledge about the health benefits of physical activity [25]. In addition, individuals with lower income levels and low socioeconomic status may have limited opportunities to receive advice from healthcare professionals regarding preventive health measures and the importance of maintaining physical fitness and activity [26]. Poor socioeconomic status has been correlated with lower compliance and adherence to clinical exercise programs, such as medical rehabilitation programs, which can be attributed to factors such as limited work flexibility, financial difficulties, and the costs associated with healthcare coverage [27].

The present study revealed a significant association between participants' health perception and their engagement in moderate-to-high levels of physical activity. Specifically, a higher number of participants with a very good health perception reported being actively involved in physical activity compared with those with good or poor health perceptions. Notably, individuals with poor health perception, particularly older adults, faced the greatest obstacles in adhering to exercise routines [28].

Furthermore, a logistic regression model was employed in our study to examine the relationship between physical activity and mood. Surprisingly, none of the mood subscales emerged as strong predictors of physical activity, except for the subscale of vigor, which exhibited substantial predictive power. These findings align with previous research conducted in Minneapolis, where a longitudinal study on 213 obese individuals demonstrated that an increase in energy expenditure was positively associated with an increase in vigor over a relatively short period of time (6 months). Moreover, exercise was found to be a significant predictor of both vigor and fatigue [29]. Similarly, Werneck and Navarro [30] explored the relationship between the physical activity level and mood in a sample of 41 adolescents (boys and girls) and discovered a beneficial effect of physical activity on total mood disorder and vigor. The study revealed that higher levels of physical activity were associated with a stronger correlation with vigor [30].

The present study employed a logistic regression model to investigate the predictive factors of physical activity, and the results indicated that self-efficacy played a substantial role in determining physical activity levels. This finding is consistent with previous research in this field. Resnick and D'Adamo [31] examined a group of 163 older adults residing in a retirement community and found a direct association between self-efficacy, negative outcome expectations, and exercise behavior [31]. Similarly, a study conducted by White et al. [32] on middle-aged and older adults over an 18-month period demonstrated that self-efficacy influenced physical activity directly as well as indirectly through outcome expectations, another construct of social cognitive theory (SCT) [32]. Across various time periods, self-efficacy consistently emerged as the strongest predictor of physical activity levels. Numerous empirical studies have emphasized the significant role of self-efficacy in adolescents' engagement in physical activity, with self-efficacy to overcome barriers to physical activity being a key predictor [33, 34]. In addition, a comprehensive review of determinants of physical activity in adolescence identified a significant and positive correlation between self-efficacy and physical activity in 28 studies [35]. It is worth noting that the relationship between self-efficacy and physical activity is complex, as other constructs of social cognitive theory, such as self-regulation and management, may mediate this effect [36]. Furthermore, research has revealed that individuals with low self-efficacy levels exhibit higher physical activity levels when they engage more frequently in preparatory behaviors [37]. In the logistic regression model used, the significant variables examined in this study, namely, education, income, good health perception, self-efficacy, and one mood subscale (vigor), collectively accounted for 11–18% of the variance.

Although this is the first study to report the relationship between mood and self-efficacy with physical activity, it has limitations. This study is observational, and hence, causation may not be established. In addition, our inability to recruit participants from refugee camps may have introduced selection bias. However, our findings are still relevant to at least a portion of refugees in Jordan and are consistent with the literature.

In conclusion, this study established statistically significant associations between gender, education level, income, health status, self-efficacy, and mood subscales with physical activity levels among Syrian and Iraqi refugees in Jordan, whereby being male and having higher education, higher income, better health, higher self-efficacy, and increased vigor correlated with higher physical activity. These findings highlight the importance of considering sociodemographic factors and psychological aspects, such as self-efficacy and mood, when addressing physical activity among refugees in Jordan. [38].

## Figures and Tables

**Table 1:** Association between physical activity and demographical factors.

Factor	Categories	*N* (%)	Low physical activity, *N* (%)	Moderate to high physical activity, *N* (%)	Chi-square value (*P* value)
Age	18–30 years	370 (35.6%)	289 (80.5%)	72 (19.5%)	1.29 (0.730)
	31–50 years	477 (46%)	383 (80.3%)	94 (19.7%)	
	51–60 years	123(11.8%)	104 (84.6%)	19 (15.4%)	
	More than 60 years	68 (6.6%)	56 (82.4%)	12 (17.6%)	

Marital status	Single	140 (13.5%)	107 (76.4%)	33 (23.6%)	4.35 (0.224)
	Married	809 (77.9%)	660 (81.6%)	149 (18.4%)	
	Divorce	38 (3.7%)	29 (76.3%)	9 (23.7%)	
	Widowed	51 (4.9%)	45 (88.2%)	6 (11.8%)	

Gender	Male	401 (38.6%)	307 (76.6%)	94 (23.4%)	8.46 (0.004)^*∗*^
	Female	637 (61.4%)	534 (83.8%)	103 (16.2%)	

Education	No official education	223 (21.5%)	208 (93.3%)	15 (6.7%)	49.19 (0.001)^*∗*^
	Primary school	306 (29.5%)	260 (85.0%)	46 (15.0%)	
	Above primary school and below secondary	278 (26.8%)	213 (76.6%)	65 (23.4%)	
	Secondary	184 (17.7%)	128 (69.6%)	56 (30.4%)	
	University	47 (4.5%)	32 (68.1%)	15 (31.9%)	

Job	Yes	134 (12.9%)	104 (77.6%)	30 (22.4%)	1.16 (0.289)
	No	904 (87.1%)	737 (81.5%)	167 (18.5%)	

Chronic diseases	No	429 (41.3%)	349 (81.4%)	80 (18.6%)	0.052 (0.872)
	Yes	609 (58.7%)	492 (80.8%)	117 (19.2%)	

Nationality	Syrian	826 (79.6%)	670 (81.1%)	156 (18.9%)	0.023 (0.474)
	Iraqi	212 (20.4%)	171 (80.7%)	41 (19.3%)	

Health	Very good	140 (13.5%)	100 (71.4%)	40 (28.6%)	10.63 (0.005)^*∗*^
	Good	625 (60.2%)	521 (83.4%)	104 (16.6%)	
	Bad	273 (26.3%)	220 (80.6%)	83 (19.4%)	

Smoking status	Yes	249 (24.0%)	206 (82.7%)	43 (17.3%)	0.623 (0.459)
	No	789 (76%)	635 (80.5%)	197 (19%)	

Enough income	Yes	101 (9.7%)	66 (65.3%)	35 (34.7%)	17.878 (0.001)^*∗*^
	No	937 (90.3%)	775 (82.7%)	162 (17.3%)	

^*∗*^ *P* value <0.05 implies statistical significance.

**Table 2:** Differences in self-efficacy and mood based on the physical activity level.

Factor	Low physical activity, mean (SD)	Moderate-to-high physical activity, mean (SD)	*t* value	*P* value
Self-efficacy	25.45 (6.10)	28.05 (6.17)	−5.36	0.001^*∗*^
Anger	7.55 (4.25)	6.59 (4.05)	2.88	0.004^*∗*^
Confusion	7.57 (4.61)	6.03 (4.60)	4.22	0.001^*∗*^
Depression	6.82 (4.28)	5.48 (4.31)	3.94	0.001^*∗*^
Fatigue	7.29 (4.16)	6.06 (4.05)	3.73	0.001^*∗*^
Tension	7.88 (4.0)	7.02 (4.24)	2.70	0.007^*∗*^
Vigor	8.96 (4.41)	6.69 (4.78)	6.38	0.001^*∗*^

^*∗*^ *P*-value <0.05 implies statistical significance.

**Table 3:** Logistic regression for physical activity.

Variables	*β*	SE	Wald test	df	*P* value	Odds ratio
Gender	0.448	0.174	6.642	1	0.010^*∗*^	1.565
No official education	−1.511	0.431	12.299	1	0.001^*∗*^	0.221
Primary school	−0.877	0.371	5.57	1	0.018^*∗*^	0.416
Above primary school and below secondary	−0.237	0.362	0.431	1	0.512	0.789
Secondary	0.070	0.372	0.035	1	0.851	1.072
Enough income	0.573	0.258	4.931	1	0.026^*∗*^	1.773
Very good health	−0.201	0.289	0.485	1	0.486	0.818
Good health	−0.524	0.210	6.223	1	0.013^*∗*^	0.592
Self-efficacy	0.33	0.15	5.046	1	0.025^*∗*^	1.034
Anger	0.028	0.032	0.770	1	0.380	1.029
Confusion	−0.026	0.029	0.757	1	0.384	0.975
Depression	−0.29	0.035	0.691	1	0.406	0.972
Fatigue	−0.34	0.031	1.236	1	0.266	0.967
Tension	0.013	0.031	0.175	1	0.675	1.013
Vigor	−0.093	0.020	21.460	1	0.001^*∗*^	0.911
Constant	−0.732	0.619	1.397	1	0.237	0.481

*β*, logistic coefficient; df, degrees of freedom; SE, standard error. ^*∗*^*P* value <0.05 implies statistical significance.

## Data Availability

The data used to support the findings of this study are available from the corresponding author upon request.
